# Co-culture of primary CLL cells with bone marrow mesenchymal cells, CD40 ligand and CpG ODN promotes proliferation of chemoresistant CLL cells phenotypically comparable to those proliferating *in vivo*

**DOI:** 10.18632/oncotarget.2939

**Published:** 2014-12-03

**Authors:** Noelia Purroy, Pau Abrisqueta, Júlia Carabia, Cecilia Carpio, Carles Palacio, Francesc Bosch, Marta Crespo

**Affiliations:** ^1^ Laboratory of Experimental Hematology, Department of Hematology, Vall d'Hebron University Hospital, Universitat Autònoma de Barcelona, Barcelona, Spain

**Keywords:** CLL, co-culture, proliferation, immunophenotype, chemoresistance, ZAP-70

## Abstract

Chronic lymphocytic leukemia (CLL) cells residing in the bone marrow (BM) and in secondary lymphoid tissues receive survival and proliferative signals from the microenvironment, resulting in persistence of residual disease after treatment. In this study, we characterized primary CLL cells cultured with BM stromal cells, CD40 ligand and CpG ODN to partially mimic the microenvironment in the proliferative centers. This co-culture system induced proliferation and chemoresistance in primary CLL cells. Importantly, co-cultured primary CLL cells shared many phenotypical features with circulating proliferative CLL cells, such as upregulation of ZAP-70 and CD38 and higher CD49d and CD62L expression. This indicates aggressiveness and capability to interact with surrounding cells, respectively. In addition, levels of CXCR4 were decreased due to CXCR4 internalization after CXCL12 stimulation by BM stromal cells. We suggest that this co-culture system can be used to test drugs and their combinations that target the proliferative and drug resistant CLL cells.

## INTRODUCTION

Chronic lymphocytic leukemia (CLL), the most prevalent B-cell malignancy in adults in Western countries, is characterized by the expansion of monoclonal mature B cells expressing CD5 and CD23 in peripheral blood (PB), secondary lymphoid tissues, and the bone marrow (BM)[1]. CLL cells had been described to accumulate as a result of a defective apoptosis, rather than of an increased proliferation[1]. This hypothesis was mainly based on the fact that most of the circulating cells are arrested in the G_0_/G_1_ phase of the cell cycle[2]. However, it was later shown that a distinct fraction of CLL cells are proliferating, being the cell birth rate 0.1% to 1% of the CLL clone per day[3]. CLL cells mainly receive proliferative signals in tissue compartments, such as lymph nodes (LN) and BM, where CLL cells form aggregates of activated, proliferating cells called “proliferation centers” or “pseudofollicles”[4]. Within this tissue microenvironment, CLL cells receive advantageous signals from accessory cells such as T cells[5], mesenchymal stromal cells[6] and nurse-like cells[7]. These signals are propagated through diverse receptors, such as CD40[8], Toll-like receptors (TLR)[9], CXCR4[10] and the B cell receptor (BCR)[11], which activate downstream signaling pathways that ultimately promote proliferation, modulate cell adhesion and chemotaxis and protect CLL cells from spontaneous and drug-induced apoptosis. Residual leukemic cells residing in these protective niches after treatment are therefore potentially contributing to minimal residual disease (MRD) persistence and to the disease relapse virtually observed in all patients after chemotherapy even after achievement of complete remission[12],[13]. Further characterization of primary CLL cells found in the proliferative niches can facilitate the discovery and study of new therapeutic targets specifically expressed by this proliferative and chemoresistant subset of CLL cells, which could ultimately help to eradicate MRD.

With the aim of better defining proliferating primary CLL cells, we characterized primary CLL cells cultured *ex vivo* in conditions mimicking the microenvironment found in the proliferative centers and compared them to proliferating subclones of CLL cells found in PB from patients with active disease, which likely represent cells that have been stimulated in the proliferation centers while residing in the LN or BM before becoming quiescent again in PB [14]. The herein described *ex-vivo* culture system induced proliferation of primary CLL cells that shared physiologic and immunophenotypic characteristics with those proliferating CLL cells found *in vivo*, providing an easily reproducible system for the ex vivo testing of new drugs specifically targeting this clinically relevant compartment of CLL cells.

## RESULTS

### Effects of BMSC, CD40 and CpG ODN in proliferation, cell cycle and viability of primary CLL cells

**Figure 1:**
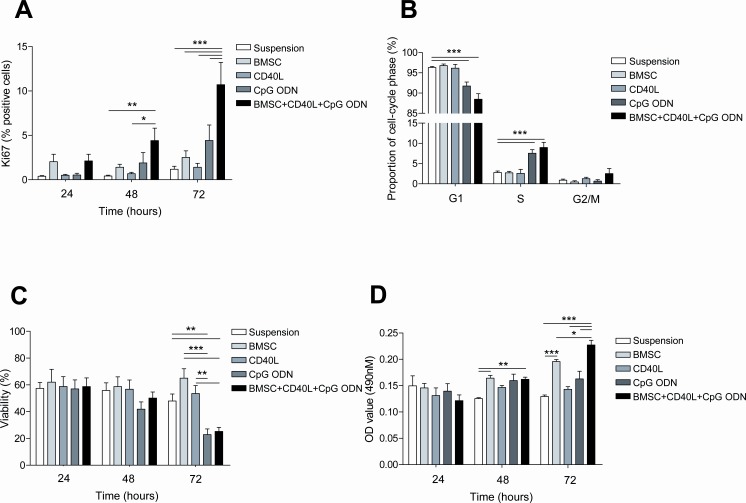
The co-culture of primary CLL cells with BMSC, CD40L and CpG ODN induces proliferation of CLL cells Primary CLL cells were cultured with BMSC, with CD40L, with CpG ODN or with BMSC, CD40L and CpG ODN. Cellular analyses were performed after 24, 48 and 72 hours of culture. (A) Mean % of Ki-67-positive cells from 8 patients was analyzed by flow cytometry. (B) Cell cycle distribution was assessed in primary CLL cells from 8 patients by PI staining by FC. (C) Viability was assessed in primary CLL cells from 8 patients by Annexin V and PI staining (D) MTS assay of co-cultured CLL cells. The mean optical density (OD) values at 490nm ± SEM from triplicates from 4 patients are depicted in the graph.

With the aim of partially ex vivo mimicking the microenvironment found in the proliferative centers, we cultured primary CLL cells in 5 different culture conditions: in suspension, co-cultured with the BMSC cell line UE6E7T-2, with soluble CD40L, with CpG ODN and with the combination of all elements: BMSC, CD40L and CpG ODN. Next, we analyzed the effects in terms of proliferation and survival after 24, 48 and 72 hours of culture. In this setting, proliferative responses, measured as increase in expression of Ki-67, were significantly observed after 48 hours in CLL cells cultured with BMSC, CD40L and CpG ODN (Figure 1A) (mean % Ki-67 positive cells: 4.41±1.41 cultured with BMSC, CD40L and CpG ODN *vs.* 0.42±0.10 in suspension, *P*<0.01) and were even higher after 72 hours when, as we had previously observed [15], the co-culture with BMSC, CD40L and CpG ODN induced a Ki-67 expression of 10.71% ± 2.51% *vs.* 1.18%±0.34% in suspension (*P*<0.001). Interestingly, when we analyzed the effect of every stimulus independently we observed that none of them increased Ki-67 expression significantly (mean % Ki-67 positive cells: 1.18±0.34 cultured in suspension vs. 2.52±0.74 cultured with BMSC vs. 1.41±0.44 cultured with CD40L vs. 4.42±1.75 cultured with CpG ODN, *P*=n.s.).

Furthermore we analyzed the ratio of CLL cells in G_1_, S and G_2_/M phases after 72 hours of culture in the above described different culture conditions. Cell cycle analysis showed that the addition of CpG ODN to CLL cells culture induced an entry into S/G_2_/M phase, this increasing with the addition of BMSC and CD40L (Figure 1B) (mean % cells in S phase with CpG ODN *vs.* suspension: 7.56±0.89 *vs.* 2.81±0.38, *P*<0.001; mean % cells in S phase with BMSC+CD40L+CpG ODN *vs.* suspension: 8.99±1.26 *vs.* 2.81±0.38, *P*<0.001; mean % cells in G_2_/M phase with BMSC+CD40L+CpG ODN *vs.* suspension: 2.54±1.27 *vs.* 0.89±0.28, *P*=n.s.).

Viability assays showed that none of the analyzed co-culture conditions induced significant changes in CLL cells viability until 72 hours. At this time point, both BMSC and CD40L alone protected primary CLL cells from spontaneous apoptosis although statistical significance was not reached (Figure 1C) (mean % of viable cells in suspension *vs.* co-cultured with BMSC *vs.* with CD40L: 47.95±5.27 *vs.* 65.05±7.02 *vs.*53.53±5.95, *P*=n.s.). In contrast, the addition of CpG ODN induced a significant decrease in the percentage of viable cells which was not restored with the addition of BMSC and CD40L (mean % of viable cells in suspension *vs.* with CpG ODN: 47.95±5.27 *vs.* 22.88±4.26, *P*<0.01; mean % of viable cells in suspension *vs.* BMSC+CD40L+CpG ODN: 47.95±5.27 *vs.* 25.22±3.11, *P*<0.01). Since we observed the higher proliferation rate concomitant with the lower percentage of alive cells, especially after 72 hours of culture, we measured metabolic activity using an MTS-based cell assay. We observed that the results were comparable to Ki-67 analysis, being the co-culture with BMSC, CD40L and CpG ODN the condition inducing the highest degree of proliferation after 72 hours. These results strongly suggest that the lower percentage of viable cells simultaneously observed with an increase in Ki-67-positive cells and metabolic activity is probably caused by exhaustion of nutrients in the culture media due to a rapid CLL cell turnover.

Finally, we did not observe any significant correlation between the induction of proliferation or survival by the co-culture with BMSC, CD40L and CpG ODN and clinical stage, CD38, ZAP-70 expression or the mutational status of IGHV genes (Table 1).

**Table 1:** Ki-67 expression and viability relative to suspension according to clinical and biological parameters

		KI-67 expression relative to suspension			Viability relative to suspension		
		Fold chango	N	T-test *p* value	%	N	T-test *p* value
**Stage**	**A**	11D5±2.42	13	0.430	62.24±11.59	12	0.673
	**B+C**	7.23±4.76	5		45.54±4.44	6	
**CD38 expression**	**≤30%**	9.70±2.46	13	0.767	54.81±9.95	14	0.222
	**>30%**	10.75±4.85	5		63.19±10.01	4	
**ZAP-70 expression**	**≤20%**	12.13±3.34	10	0.315	53.29±10.56	12	0.325
	**>20%**	7.32±2.34	8		63.44±11.98	6	
**IGFiV status**	**mutated**	5.08±3.21	3	0.811	61.08±28.15		0.727
	**unmutated**	7.7432.95	10		65.78±13.26	9	

### Immunophenotypical differences between resting and proliferative primary CLL cells from PB

**Figure 2:**
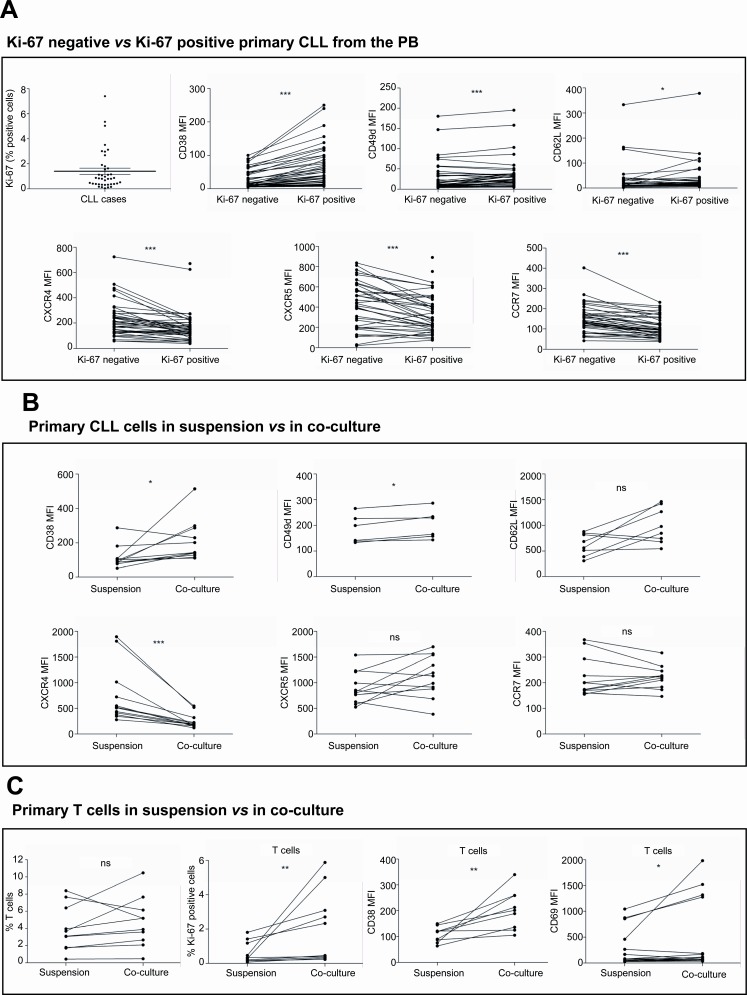
The co-culture of primary CLL cells with BMSC, CD40L and CpG ODN promotes an immunophenotype comparable to that from proliferating CLL cells found in PB (A) PBMC from 40 patients diagnosed with CLL were used to analyze by FC the differential expression of CD38, CD49d, CD62L, CXCR4, CXCR5 and CCR7 in Ki-67-negative *vs.* positive CLL cells. (B) Primary CLL cells from 12 patients were cultured in suspension or in co-culture with BMSC, CD40L and CpG ODN for 48 hours and the expression ofCD38, CD49d, CD62L, CXCR4, CXCR5 and CCR7 were analyzed. (C) PBMC from 10 patients diagnosed with CLL were cultured in suspension or in co-culture with BMSC, CD40L and CpG ODN for 48 hours and the percentage of T cells and their expression levels of Ki-67, CD38 and CD69 were analyzed. (*P<0.05, **P<0.01, ***P<0.001, ns: non significant, paired T-test).

With the aim of comparing primary CLL cells cultured *ex vivo* in conditions mimicking the microenvironment found in the proliferative centers with proliferating subclones of CLL cells found in PB from patients with active disease, we initially analyzed proliferating CLL cells from 40 patients diagnosed with CLL. For this, we analyzed by FC the differential expression of CD38, CD49d, CD62L and the chemokine receptors CXCR4, CXCR5 and CCR7 in Ki-67 positive *vs.* Ki-67 negative CLL cells (Figure 2A). Mean percentage of Ki-67 expression in CLL samples was 1.40±0.26 (range, 0.05-7.41). Ki-67-positive CLL cells showed higher expression levels of CD38 (mean MFI of CD38 expression in Ki-67 positive cells *vs.* Ki-67 negative cells: 57.46±8.43 *vs.* 25.41±3.83, *P*<0.001). The expression levels of integrin CD49d and selectin CD62L were also higher in Ki-67 positive CLL cells than in Ki-67 negative cells (mean MFI of CD49d expression in Ki-67 positive cells *vs.* Ki-67 negative cells: 44.92±10.15 *vs.* 39.53±10.43, *P*<0.01; mean MFI of CD62L expression in Ki-67 positive cells *vs.* Ki-67 negative cells: 37.87±10.03 *vs.* 31.48±9.41, *P*<0.05) while the expression levels of all the chemokine receptors analyzed were significantly lower (mean MFI of CXCR4 expression in Ki-67 positive cells *vs.* Ki-67 negative cells: 172.3±20.03 *vs.* 223.2±22.35, *P*<0.001; mean MFI of CXCR5 expression in Ki-67 positive cells *vs.* Ki-67 negative cells: 343.4±31.37 *vs.* 428.7±38.18, *P*<0.001; mean MFI of CCR7 expression in Ki-67 positive cells *vs.* Ki-67 negative cells: 110.1±8.07 *vs.* 149.2±10.65, *P*<0.001)(Figure 2A).

### The co-culture of primary CLL cells with BMSC, CD40L and CpG ODN promotes an immunophenotype comparable to that from proliferating CLL cells found in PB

As described above, we observed that the co-culture of primary CLL cells in conditions mimicking the microenvironment of the proliferative centers induced the proliferation of CLL cells in terms of Ki-67 expression, MTS-based cell proliferation assay and cell cycle entry. In order to compare the immunophenotype of proliferating CLL cells found *in vivo* with the ex-vivo stimulated CLL cells, we analyzed the modulation of the expression of CD38, CD49d, CD62L, CXCR4, CXCR5 and CCR7 in primary CLL cells after 48 hours of co-culture as compared to CLL cells in suspension (Figure 2B). Primary CLL cells in co-culture showed an increase in the expression of CD38, CD49d and CD62L (mean MFI of CD38 expression in co-culture *vs.* in suspension: 151.9±42.56 *vs.* 60.16±4.79, *P*<0.05; mean MFI of CD49d expression in co-culture *vs.* in suspension: 202.8±22.8 *vs.* 184.3±22.48, *P*<0.05; mean MFI of CD62L expression in co-culture *vs.* in suspension: 993.9±123.7 *vs.* 626.0±76.49, *P*=n.s.). In addition, co-culture of CLL cells induced a downregulation of the expression level of CXCR4 (mean MFI of CXCR4 expression in co-culture *vs.* in suspension: 247.6±41.23 *vs.* 741.0±160.3, *P*<0.001) while CXCR5 and CCR7 expression levels were not significantly modulated (mean MFI of CXCR5 expression in co-culture *vs.* in suspension: 1122±121.0 *vs.* 906.6±94.32, *P*=n.s.; mean MFI of CCR7 expression in co-culture *vs.* in suspension: 221.4±13.79 *vs.* 225.0±23.43, *P*=n.s.).

To determine whether the co-culture of PBMC from patients with CLL with BMSC, CD40L and TLR9L also stimulated T cells we analyzed the expression of Ki-67, CD38 and CD69 expression in CD3+ mononucleated cells (Figure 2C). Taking into account that only samples with at least 85% of CLL cells were included in our study, we observed a mean percentage of T cells of 4.02±0.84 at baseline, which was not significantly altered after 48 hours in co-culture. However, the co-culture did significantly increase Ki-67, CD38 and CD69 expression in T cells (Figure 2C) indicating that T cells were also activated under these co-culture conditions.

### The co-culture of primary CLL cells with BMSC, CD40L and CpG ODN preferentially increases the proliferative CXCR4^dim^CD5^br^ compartment

**Figure 3:**
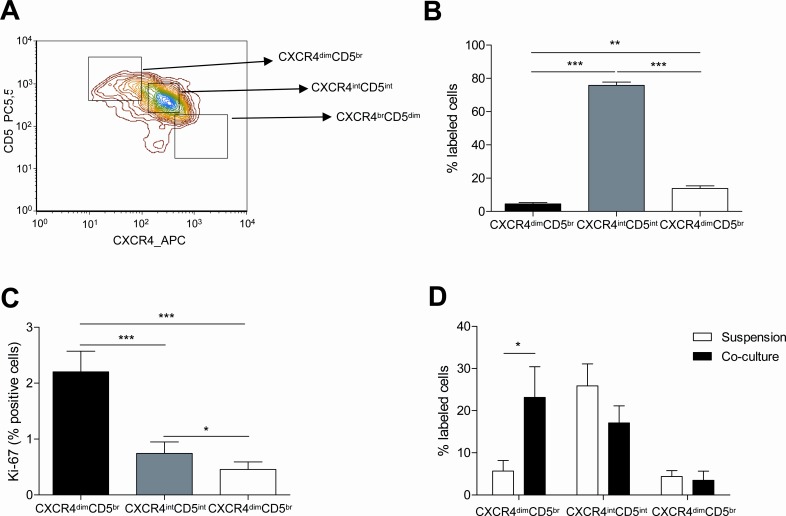
The proliferative CXCR4^dim^CD5^br^ compartment of CLL cells is promoted by the co-culture with BMSC, CD40L and CpG ODN PBMC from 40 patients diagnosed with CLL were used to analyze the expression of CXCR4 and CD5 by FC. (A) Representative contour plot of CXCR4 and CD5 expression by CLL cells from one patient. (B) Mean percentage ± SEM of CLL cells in the three compartments defined by CXCR4 and CD5 densities are depicted in the graph (**P<0.01, ***P<0.001, one-way ANOVA). (C) Mean percentage ± SEM of Ki-67 expression in the three compartments (*P<0.05, **P<0.01, ***P<0.001, one-way ANOVA). (C) Primary CLL cells from 7 patients cultured in suspension or in co-culture with BMSC, CD40L and CpG ODN for 48 hours were used to analyze the expression of CXCR4 and CD5. (*P<0.05, two-way ANOVA, Bonferroni's post-test. Graph shows mean ± SEM).

We also characterized the proliferative and resting compartments of CLL cells found in PB using differences in the expression of CD5 and CXCR4 by FC as previously defined by Calissano, C et al[16] (Figure 3A). The percentage of CLL cells falling into each compartment was similar to the previously observed distribution[16] (Figure 3B) (mean % CLL cells: 4.52±0.82 in CXCR4^dim^CD5^bright^ fraction *vs.* 75.78±2.05 in CXCR4^int^CD5^int^ fraction *vs.* 13.83±1.53 in CXCR4^bright^CD5^dim^ fraction, *P*<0.001). As expected, the proliferative compartment defined as the CXCR4^dim^CD5^bright^ fraction was significantly enriched in Ki-67 positive cells (Figure 3C) (mean % Ki-67 positive cells: 3.28±1.12 in CXCR4^dim^CD5^bright^ fraction *vs.* 2.63±0.85 in CXCR4^int^CD5^int^ fraction, *P*=n.s.; *vs.* 0.36±0.21 in CXCR4^bright^CD5^dim^ fraction, *P*<0.01). As we previously reported[15], co-culturing CLL cells for 48 hours induced an increase in the percentage of CLL cells within the CXCR4^dim^CD5^bright^compartment (Figure 3D) whereas the proportion of CXCR4^int^CD5^int^ and CXCR4^bright^CD5^dim^ cells was not significantly affected (mean % CXCR4^dim^CD5^bright^ cells: 23.16±7.25 in co-culture *vs.* 5.67±2.52 in suspension, *P*<0.05).

### The co-culture of primary CLL cells with BMSC, CD40L and CpG ODN induces ZAP-70 expression

**Figure 4:**
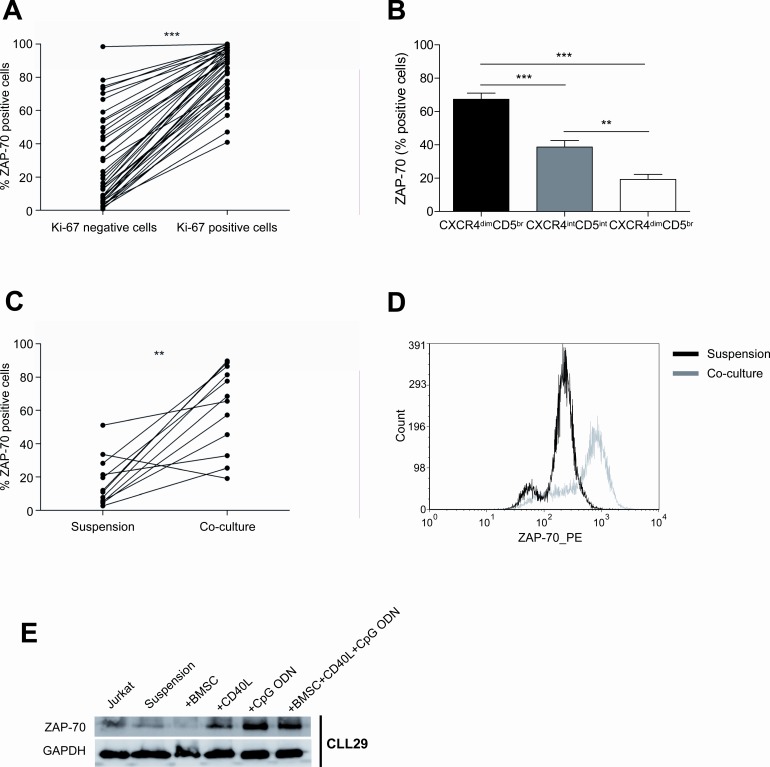
The co-culture of primary CLL cells with BMSC, CD40L and CpG ODN markedly enhances ZAP-70 expression (A) PBMC from 40 patients diagnosed with CLL were used to analyze ZAP-70 expression in Ki-67 negative vs. positive CLL cells (***P<0.001, paired T-test). (B) ZAP-70 expression in CXCR4 and CD5 compartments of CLL cells from PBMC from 40 patients. (**P<0.01, ***P<0.001, one-way ANOVA. Graph shows mean ± SEM). (C) Primary CLL cells from 12 patients were cultured in suspension or in co-culture with BMSC, CD40L and CpG ODN for 48 hours and the expression level of ZAP-70 was analyzed by FC (**P<0.01, paired T-test). (D) One representative histogram of primary CLL cells from one patient after 48 hours in suspension or in co-culture. (E) One representative immunoblot analysis of ZAP-70 expression.

Among the diverse molecular pathways of crosstalk between CLL cells and their microenvironment, BCR signaling has been recognized as one of the most important [17][18]. The expression of the protein tyrosine kinase ZAP-70 has been associated with increased BCR signaling in CLL [19] which translated into increased proliferation and migrative capacity of ZAP-70 positive subclones, based on *in vitro* and *in vivo* data [20],[21],[22],[23]. Clinically, ZAP-70 expression has been correlated with IgVH mutational status, disease progression and survival[24]. Therefore, we hypothesized that ZAP-70 expression could be upregulated in proliferating CLL subclones. In order to test this, we assessed ZAP-70 expression in CLL cells from PB according to Ki-67 expression and subsequently in primary CLL cells co-cultured in proliferative conditions. Firstly, we observed that the Ki-67 positive fraction of CLL cells from the PB was significantly enriched in ZAP-70 positive cells (Figure 4A) (mean % of ZAP-70 expression: 83.93±2.40 in Ki-67 positive cells *vs.* 29.22±4.20 in Ki-67 negative cells, *P*<0.001). We also determined ZAP-70 expression according to CXCR4 and CD5 expression and interestingly, we observed that the proliferative CXCR4^dim^CD5^bright^ fraction was also enriched in ZAP-70 positive cells (Figure 4B) (mean % ZAP-70 positive cells: 67.35±3.66 in CXCR4^dim^CD5^bright^ fraction *vs.* 38.71±3.87 in CXCR4^int^CD5^int^ fraction, *P*<0.001; *vs.* 19.29±3.04 in CXCR4^bright^CD5^dim^ fraction, *P*<0.001). In order to elucidate if signals from the microenvironment could directly modulate the expression of ZAP-70, we cultured primary CLL cells in suspension or co-cultured with BMSC, CD40L and CpG ODN for 48 hours and observed that the percentage of ZAP-70 positive cells was significantly increased (Figure 4C and 4D) (mean % ZAP-70 positive cells: 61.50±7.33 in co-culture *vs.* 16.91±4.23 in suspension, *P*<0.01). This was further confirmed by western blot, where we also observed ZAP-70 up-regulation in primary CLL cells co-cultured with BMSC, CD40L and CpG ODN for 48 hours. To clarify which of these stimuli contributed to ZAP-70 up-regulation, we cultured primary CLL cells with BMSC, CD40L or CpG ODN separately, and assessed ZAP-70 expression by western blot. We observed a marked ZAP-70 up-regulation as a consequence of either CD40 or TLR9 stimulation alone (Figure 4E).

### Primary CLL cells in co-culture develop marked chemoresistance to treatment with fludarabine and bendamustine

**Figure 5:**
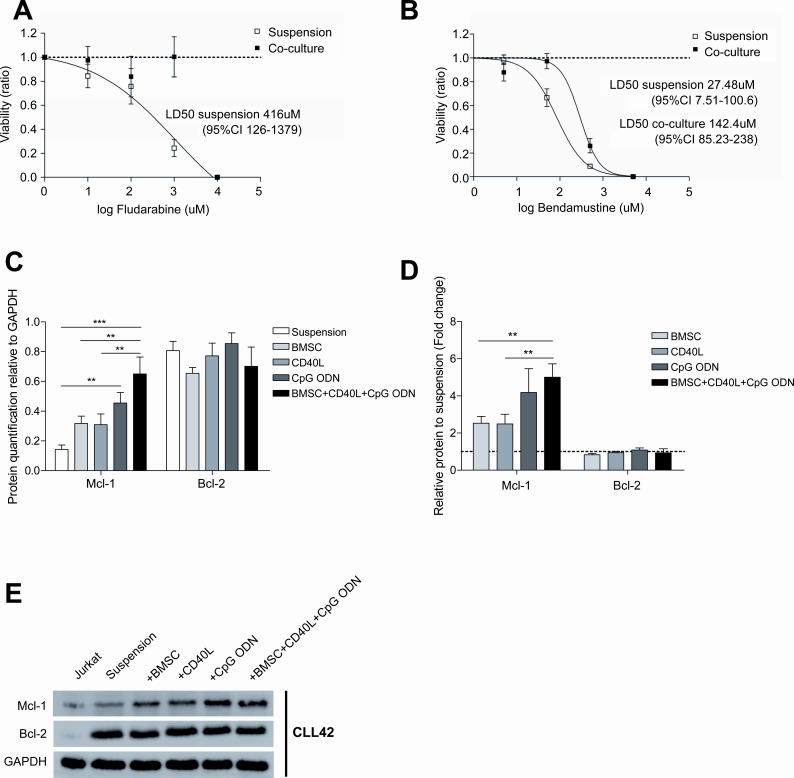
Co-cultured CLL cells display a marked chemoresistance to fludarabine and bendamustine treatment Primary CLL cells from 7 patients were cultured for 48 hours in suspension or in co-culture with BMSC, CD40L and CpG ODN; subsequently, increasing doses of fludarabine and bendamustine were added for additional 24 hours. LD50 curves for fludarabine (A) and bendamustine (B) are plotted on a logarithmic scale. (C) Quantification of Mcl-1 and Bcl-2 expression analyzed by western blot relative to GAPDH. Each bar represents the mean ± SEM from 7 patients (**P<0.01, ***P<0.001, two-way ANOVA, Bonferroni's post-test). (D) Mcl-1 and Bcl-2 expression relative to primary CLL cells in suspension. Each bar represents the mean ± SEM from 7 patients (**P<0.01, two-way ANOVA, Bonferroni's post-test). (E) One representative immunoblot analysis of Mcl-1 and Bcl-2 expression.

The microenvironment found in the proliferative centers has been shown to provide direct pro-survival signals and to protect CLL cells from the effect of chemotherapeutical agents[7],[25]. Consequently, this proliferative and chemoresistant compartment of CLL cells has been hypothesized to be potentially responsible for MRD persistence and disease relapse. In this regard, to evaluate the role of co-culture on the chemoresistance of primary CLL cells, we co-cultured primary CLL cells for 48 hours and subsequently treated them with increasing doses of fludarabine or bendamustine for additional 24 hours. Interestingly, as we previously described[15], the co-culture of CLL cells in these conditions inhibited at such extend the capacity of fludarabine to induce apoptosis that it was not possible to calculate its LD_50_, whereas LD_50_ of fludarabine in CLL cells in suspension was 416μM (95% CI 125.5-1379) (Figure 5A). For CLL cells treated with bendamustine, LD_50_ of CLL cells in proliferative conditions was 5.18 times higher than for CLL cells in suspension (Figure 5B).

To determine the molecular mechanisms related to this co-culture-induced chemoresistance we analyzed the expression of the anti-apoptotic proteins Mcl-1 and Bcl-2, which are up-regulated in CLL cells and provide them with protection against apoptosis induced by chemotherapy[26],[27]. After 48 hours of co-culture only the expression of Mcl-1 was significantly increased (Figures 5C, 5D and 5E), further highlighting its role in CLL resistance to chemotherapy.

## DISCUSSION

Compelling evidence suggests that the crosstalk between CLL cells and accessory cells in the BM and/or lymphoid tissue microenvironments plays a relevant role in the natural history of CLL by promoting tumoral cell survival, proliferation and drug resistance (Reviewed in Burger et al[4]). Therefore, further characterization of the different cells and stimuli participating in this process is of great interest in order to define new therapeutic targets aimed at interfering with this favorable condition. Based on these considerations, the aim of this study was to further characterize this proliferative and chemoresistant CLL compartment by firstly studying the phenotypic characteristics of proliferating CLL cells found in the PB of patients. Subsequently, we compared their features with those found after co-culture in conditions partially mimicking the microenvironment from the proliferation centers. This allowed us to describe an easily reproducible *ex vivo* system that will facilitate the study of this crucial CLL cell compartment and consequently, the discovery of new therapeutic targets.

We co-cultured primary CLL cells with BMSC since they have been demonstrated to support the survival of CLL cells from both spontaneous and drug-induce apoptosis [6],[7],[28],[29]. Moreover, it has been found that BMSC can activate resting CLL cells to increase their expression of CD38, as well as promote activation of CD71, CD69, CD25 and CD70[30]. Based on evidences from *in vitro* experiments with CD40L that indicate the importance of T cells with regards to CLL cell proliferation and survival[31],[32],[33][8],[34],[5], we added soluble CD40L to our co-culture system. Finally, we used the TLR9 agonists CpG ODN to stimulate primary CLL cells. Toll-like receptors have been described as potent activators of CLL cell proliferation, cytokine production and upregulation of costimulatory molecules involved in B cell-T cell interaction such as CD40, CD80, CD86, CD54 MHC class I and CD58[35][36][37]. Simultaneous stimulation with CD40/CpG ODN has been studied previously in CLL cells, observing enhanced immunogenicity[35] and distinctive proliferation in IGHV unmutated CLL cells correlating with the development of drug resistance[36].

In addition, we observed a modulation of the immunophenotype of CLL cells that was remarkably comparable to that from proliferating CLL cells found in PB of patients. These phenotypical changes indicate that these CLL cells become activated and consequently, more aggressive as shown by enhanced CD38 and ZAP-70 expression, and more likely to interact with other cells as shown by enhanced CD49d and CD62L expression. Regarding chemokine receptors, we observed downregulation of CXCR4, CXCR5 and CCR7 in proliferating CLL cells from the PB, probably indicating a recent recirculation from BM or lymphoid tissue. In the ex vivo co-culture system this was partially reproduced: CXCR4 expression was downregulated, while CXCR5 and CCR7 expression remained otherwise stable. This might be explained by the fact that our co-culture system included BMSC which produce the CXCR4 ligand CXCL12[38], but not MSC from secondary lymphoid tissues which are the responsible for CXCL13 and CCL19 production, ligands for CXCR5 and CCR7 respectively[39],[40],[41]. Additionally, the co-culture also resulted in stimulation of T cells from patients with CLL, which probably contributed to the activation of CLL cells. Besides its prognostic value, ZAP-70 expression is linked to activation of CLL cells[19],[22],[42] and its expression correlates with that of Ki-67 in CLL patients[21]. ZAP-70 expression has been clinically and biologically linked to aggressive features in CLL, but its regulation is still largely unknown; interestingly, we showed that the proliferative fraction of CLL cells from the PB was markedly enriched in ZAP-70 positive cells, and that the herein described co-culture system induced the expression of ZAP-70 in proliferating cells. Both CD40 and TLR9 stimulation independently were able to provoke an increase in the expression of ZAP-70, although CD40L alone was not enough to induce proliferation. Functionally, we observed that this co-culture system promoted the proliferation of chemoresistant CLL cells, as shown in fludarabine and bendamustine-treated primary CLL cells, and that was accompanied by the induction of the expression of the anti-apoptotic proteins Mcl-1. This is in line with previous studies using not only stromal cells [28],[29],[43] but also CD40 [8],[44] and TLR9 [9] stimulation in CLL cells culture systems[15]. In summary, our findings demonstrate that the co-culture of CLL cells with BMSC, CD40L and CpG ODN promotes the proliferation of chemoresistant primary CLL cells with a phenotype comparable to that from the circulating proliferative CLL cell. CLL therapy is heading towards targeted therapies as new and more effective drugs are emerging. This co-culture system has already been used for testing the survivin inhibitor YM155 which resulted specifically active to target the proliferative and drug resistant CLL cell compartment[15]. Therefore, this study provides a model for a co-culture system which might serve as a basis for testing these new drugs, particularly in combination, to better select the most active treatment for this disease.

## METHODS

### Isolation and culture of primary CLL cells

Peripheral blood mononuclear cells (PBMC) from 64patients diagnosed with CLL were obtained by Ficoll-Paque Plus (GE Healthcare, Buckinghamshire, United Kingdom) density gradient and subsequently criopreserved until analysis. Only samples with ≥85% of CLL cells (CD19^+^/CD5^+^ cells, as assessed by flow cytometry (FC)) were included in the study. Written informed consent was obtained from all patients in accordance with the Declaration of Helsinki and the study was approved by the local clinical investigation ethical committee.

### Cell lines

The UE6E7T-2 human bone marrow stromal cells (BMSC) cell line was obtained from Riken Cell Bank (Ibaraki, Japan). Cells were cultured at 37°C in 5% CO_2_ atmosphere in Dulbecco's Modified Eagle Medium (DMEM; Gibco, Carbbad, CA, USA) supplemented with 2mM L-glutamine, 10% heat-inactivated fetal bovine serum (FBS) and 50μg/mL penicillin/streptomycin. The T cell acute lymphoblastic leukemia cell line Jurkat was obtained from ATCC and was cultured in RPMI 1640 medium supplemented with 2mM L-glutamine, 10% heat-inactivated FBS and 50μg/mL penicillin/streptomycin.

### Co-culture conditions

BMSC were seeded at a concentration of 1.5×10^4^ cells/mL in 24-well plates and incubated for 24 hours to allow cells to adhere. CLL cells were cultured at a ratio of 100:1 (1.5 ×10^6^cells/mL) on confluent layers of BMSC in RPMI medium. Cells were stimulated with 1μg/mL CD40L (Peprotech, London, United Kingdom) and/or 1.5μg/mL CpG ODN (ODN2006; Invivogen, San Diego, CA, USA) when indicated. CLL cells were harvested by gently washing off, leaving the adherent stromal cell layer intact.

### Flow cytometry

Intracellular staining of Ki-67 was performed using a fluorescein isothiocyanate (FITC)-labeled antibody against Ki-67 (Becton Dickinson, Franklin Lakes, NJ, USA) after fixation and permeabilization using the BD Intrasure kit (Becton Dickinson) following the manufacturer's instructions. Surface staining of cells was performed using the following monoclonal antibodies conjugated with the indicated fluorochrome: CD19-phycoerythrin (PE) and CD5-allophycocyanine (APC) (Becton Dickinson). To characterize the phenotype of proliferative and resting compartments of CLL cells,we used the following antibodies: CD19-energy coupled dye (ECD), CD5-phycoerythrincyanine 5.5 (PC5.5) (Beckman Coulter, Brea, CA, USA), CD3-PE-cyanine 7 (Cy7), CXCR4-APC, CXCR5-APC, CCR7-APC, CD49d-APC, CD62L-APC, Ki-67-FITC (Becton Dickinson), and CD38-PE (EBioscience, San Diego, CA, USA). The rates of T cell activation and proliferation were analyzed by determining the expression of Ki-67, CD69 and CD38 in CD3+ cellsusing the following antibodies: Ki-67-FITC, CD38-PE (EBioscience), CD5-PC5.5 (Beckman Coulter), CD3-PE-Cy7 and CD69-APC (Becton Dickinson). Cells were acquired in a Navios^TM^ cytometer (Beckman Coulter) and the results were analyzed using the FCS Express 4 software (De Novo Software, Los Angeles, CA, USA).

### Cell proliferation assay

Cell proliferation was measured using the CellTiter96^TM^ Cell Proliferation Assay (Promega, Madison, WI, USA) which uses the cellular conversion of MTS tetrazolium compound into a colored formazan product. A total of 7.5 ×10^4^ CLL cells per well were seeded in a 96-well plate in 100μL of RPMI and 20μL of MTS. Plates were incubated for 1 hour at 37°C and absorbance was measured in a plate reader at 490nm.

### Cell cycle analysis

Propidium iodide (PI) was used to determine each phase of the cell cycle according to the DNA content of CLL cells. For this, cells were resuspended in ice-cold 70% ethanol and incubated at −20°C for 30 minutes. Cells were then washed twice with phosphate buffered saline (PBS) and resuspended in PBS containing 38nM sodium citrate, 10mg/mL ribonuclease A and 1μg/μL PI. Cells were finally incubated at 37°C for 30 minutes and subsequently analyzed by FC.

### Western blot analysis

Whole cell protein extracts were prepared from 4.5×10^6^cells using 50μL lysis buffer containing 20mM Tris(hydroxymethyl)aminomethane (Tris) pH 7.4, 1mM EDTA, 140mM NaCl, 1% NP-40 supplemented with 2mM sodium vanadate and protease inhibitor cocktail (Sigma-Aldrich, San Louis, MO, USA) for 1 hour at 4°C. Protein concentration was determined using the Bio-Rad protein assay (Bio-Rad, Hercules, CA, USA). Equal amounts of denatured protein were resolved by 10% SDS-PAGE and transferred to Immobilon-P membranes (Millipore, Bedford, MA, USA). Membranes were blocked for 1 hour at room temperature (RT) in 5% milk/TBS-T. Membranes were incubated overnight at 4°C with primary antibodies against ZAP-70, Mcl-1 and Bcl-2 (Santa Cruz Biotechnologies, Dallas, TX, USA), and GAPDH (Abcam, Cambridge, United Kingdom). Immunodetection was done with the corresponding IgG HRP-linked secondary antibodies (Dako North America, Glostrup, Denmark), and the ECL chemiluminescence detection system (GE Healthcare). Chemiluminescent images were acquired with the LAS-4000 system (Buckinghamshire, United Kingdom). Protein expression was subsequently quantified using the Image J 1.46r software (National Institutes of Health).

### Reagents

Fludarabine and bendamustine (Sigma, St Louis, MO, USA) were dissolved in DMSO and stored at −20°C.

### Assessment of apoptosis

Apoptosis was assessed by analyzing the binding of annexin V-FITC and the incorporation of PI by FC. Annexin V/PI double negative cells were considered viable cells. Staining was performed according to the manufacturer's instructions using the annexin V-FITC apoptosis detection kit (Bender Medsystems, Vienna, Austria).

### Statistical analysis

Results are expressed as the mean ± standard error of the mean (SEM) of at least three independent experiments. The statistically significant difference between groups was analyzed using the Mann-Whitney test or one or two-way ANOVA (*t* test), and *P*<0.05 was considered significant. Lethal dose 50 (LD_50_) values were calculated with GraphPad Prism software version 5.0 (San Diego, CA, USA). Analyses were performed using the biostatistics software package SPSS version 17 (IBM, Chicago, IL, USA). Results were graphed with GraphPad Prism software.
